# Maternal dietary selenium intake is associated with increased gestational length and decreased risk of preterm delivery

**DOI:** 10.1017/S0007114519002113

**Published:** 2019-12-23

**Authors:** Malin Barman, Anne Lise Brantsæter, Staffan Nilsson, Margaretha Haugen, Thomas Lundh, Gerald F. Combs, Ge Zhang, Louis J. Muglia, Helle Margrete Meltzer, Bo Jacobsson, Verena Sengpiel

**Affiliations:** 1 Department of Obstetrics and Gynaecology, Institute of Clinical Sciences, University of Gothenburg, S-416 85 Gothenburg, Sweden; 2 Department of Biology and Biological Engineering, Food and Nutrition Science, Chalmers University of Technology, S-412 96 Gothenburg, Sweden; 3 Division of Infection Control, Environment and Health, Norwegian Institute of Public Health, N-0213 Oslo, Norway; 4 Department of Mathematical Sciences, Chalmers University of Technology, S-412 96 Gothenburg, Sweden; 5 Department of Laboratory Medicine, Institute of Biomedicine, University of Gothenburg, S-405 30 Gothenburg, Sweden; 6 Division of Occupational and Environmental Medicine, Department of Laboratory Medicine, Lund University, S-221 00 Lund, Sweden; 7 Jean Mayer USDA Human Nutrition Research Center on Aging, Tufts University, Boston, MA 02111, USA; 8 Division of Human Genetics and Perinatal Institute, Cincinnati Children’s Hospital Medical Center and Department of Pediatrics, University of Cincinnati College of Medicine, Cincinnati, OH 45267, USA; 9 Department of Genetics and Bioinformatics, Domain of Health Data and Digitalisation, Institute of Public Health, N-0213 Oslo, Norway; 10 Department of Obstetrics and Gynaecology, Sahlgrenska University Hospital/Östra, S-413 45 Gothenburg, Sweden

**Keywords:** Preterm delivery, Gestational length, Selenium, Norwegian Mother, Father and Child Cohort Study, MoBa, Dietary selenium intake, Selenium status

## Abstract

The first positive genome-wide association study on gestational length and preterm delivery showed the involvement of an Se metabolism gene. In the present study, we examine the association between maternal intake of Se and Se status with gestational length and preterm delivery in 72 025 women with singleton live births from the population-based, prospective Norwegian Mother, Father and Child Cohort Study (MoBa). A self-reported, semi-quantitative FFQ answered in pregnancy week 22 was used to estimate Se intake during the first half of pregnancy. Associations were analysed with adjusted linear and Cox regressions. Se status was assessed in whole blood collected in gestational week 17 (*n* 2637). Median dietary Se intake was 53 (interquartile range (IQR) 44–62) µg/d, supplements provided additionally 50 (IQR 30–75) µg/d for supplement users (*n* 23 409). Maternal dietary Se intake was significantly associated with prolonged gestational length (*β* per sd = 0·25, 95 % CI, 0·07, 0·43) and decreased risk of preterm delivery (*n* 3618, hazard ratio per sd = 0·92, 95 % CI, 0·87, 0·98). Neither Se intake from supplements nor maternal blood Se status was associated with gestational length or preterm delivery. Hence, the present study showed that maternal dietary Se intake but not intake of Se-containing supplements, during the first half of pregnancy was significantly associated with decreased risk of preterm delivery. Further investigations, preferably in the form of a large randomised controlled trial, are needed to elucidate the impact of Se on pregnancy duration.

Preterm delivery (PTD; delivery before completing 37 weeks of gestation) affects 5–13 % of all pregnancies worldwide, though the incidence varies a lot even between high-income countries^(^^1^^–^^3^^)^. It is the leading cause of neonatal morbidity and mortality. There is no way of predicting or preventing spontaneous PTD, but it is urgent to identify modifiable factors influencing the prevalence of PTD and to understand the pathways that regulate the timing of birth.

Our research group recently performed a genome-wide association study regarding gestational length and PTD in a discovery cohort of 43 568 women of European ancestry^(^^4^^)^. Four loci achieved genome-wide significance: the eukaryotic elongation factor, selenocysteine transfer RNA-specific (*EEFSEC*) gene was significant for both gestational length and risk of PTD. These associations were confirmed in three Nordic cohorts, among others the Norwegian Mother and Child Cohort Study (MoBa).

The *EEFSEC* gene codes for the protein EEFSEC, which is an elongation factor necessary for the co-translational incorporation of Se into the amino acid selenocysteine and thus selenoproteins. Se is an essential trace element. Its biological function is transferred via some twenty-five selenoproteins, and Se deprivation limits the syntheses of these proteins. Selenoproteins, such as the glutathione peroxidases and the thioredoxin reductases, have important cellular homeostatic functions in maintaining redox status and antioxidant defence as well as in modulating inflammatory responses^(^^5^^)^. The Se-containing 5’deiodinases, which are required for thyroid hormone activity, are important in the regulation of growth and energy metabolism^(^^6^^)^. Redox status, inflammation and regulation of energy metabolism have been linked to the parturition process and risk of PTD^(^^7^^–^^10^^)^. The identification of the selenocysteine pathway in PTD suggests that maternal Se status may be important. The fact that Se deficiency is prevalent in Malawi, the country with the highest frequency of PTD^(^^11,12)^, further supports the hypothesis. The few existing studies on Se metabolism with regard to gestational length present contradicting results. Data from the National Birth Defects Prevention Study on 5738 deliveries (471 PTD cases) found no relationship between Se intake before pregnancy, measured in retrospect, and the risk of PTD ^(^^13^^)^. Similarly, a small observational study (*n* 233) in the USA found no association between Se concentration in plasma and PTD^(^^14^^)^. However, the study found that women delivering preterm (*n* 107) had higher estimated Se intake during early pregnancy compared with women delivering at term (*n* 126)^(^^14^^)^. A third observational study found that women who delivered preterm (*n* 60) on the contrary had lower serum Se concentration at gestational week 12 compared with those who delivered at term (*n* 1069)^(^^15^^)^.

The aim of the present study is to examine the association between self-reported maternal dietary intake of Se during the first half of pregnancy and maternal Se status as indicated by the whole-blood concentration of Se at week 17 and two outcomes, gestational length and spontaneous PTD, in the large, prospective population-based MoBa.

## Subjects and methods

### Study population

The present study is based on MoBa, an ongoing prospective population-based pregnancy cohort administered by the Norwegian Institute of Public Health^(^^16^^)^. Participants were recruited from all over Norway from 1999 to 2008, with a participation rate of 41 %. The cohort now includes 114 500 children, 95 200 mothers and 75 200 fathers^(^^16^^)^.

**Fig. 1.**
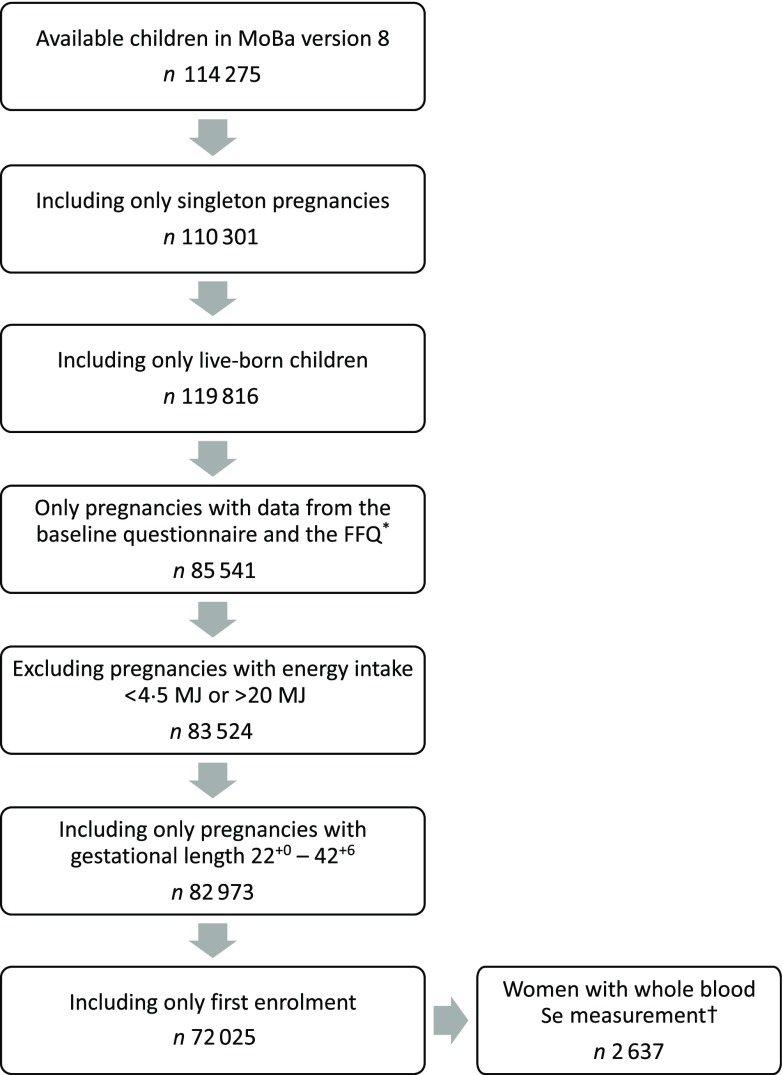
Flow chart over the study population. ^*^ The present FFQ was not used before 2002, explaining the drop of numbers from boxes 3 to 4. † Selenium was measured in *n* 2982 women. MoBa, Norwegian Mother, Father and Child Cohort Study.

Pregnant women were invited by postal invitation in connection with the routine ultrasound screening offered free of charge to all women in gestational weeks 17–19. They were asked to answer three questionnaires during their pregnancies and to donate blood and urine samples at the time of ultrasound screening. Participants were followed up regularly with questionnaires after delivery. The present study is based on information from the first questionnaire (Q1) about general health status and lifestyle, which was filled out around gestational weeks 15–17, and the semi-quantitative FFQ filled out around gestational week 22. Pregnancy and birth records from the Medical Birth Registry of Norway (MBRN) were linked to the MoBa database^(^^16^^)^. MBRN is a national health registry containing information about all births in Norway. The present study is based on version 8 of the quality-assured data files released for research in 2014. Only women with singleton live births after gestational week 22^+0^ and women with valid estimates of total energy intake (between 4·5 and 20 MJ) were included. Women who participated in the cohort with more than one pregnancy were included only with their first enrolled pregnancy. Fig. 1 outlines the selection of the study population.

### Selenium intake

Intake of Se (µg/d) from food and dietary supplements was estimated based on self-reported food and supplement intake in the MoBa (FFQ). The MoBa FFQ is a semi-quantitative instrument designed to record dietary habits and intakes of dietary supplements during the first 4–5 months of pregnancy, that is, from the start of pregnancy until around gestational week 22^(^^17^^)^. The FFQ includes questions about the intake of 255 food items or dishes. The FFQ has been validated in an MoBa subpopulation (*n* 119) using a 4-d weighed food diary and biological markers in blood and urine as reference measures^(^^18^^,^^19^^)^, showing that the MoBa FFQ is a valid tool for assessing dietary intake of energy, nutrients and food in the first 4–5 months of pregnancy.

The present version of the FFQ was used from March 2002 throughout the recruitment period. Completed questionnaires were optically read, and the consumption frequencies were converted into food amounts (g/d) using standard Norwegian portion sizes^(^^17^^)^. Intake of Se and other nutrients from food was based on FoodCalc^(^^20^^)^ and the Norwegian food composition table^(^^21^^)^. Se occurring naturally in foods is thought to be mostly selenomethionine.

The participants were asked to report the use of dietary supplements by writing name and brand as well as the frequency and amount. To calculate nutrient intake from supplements, a database of more than 1000 dietary supplements was created based on the producers’ declared nutrient content information^(^^22^^,^^23^^)^. Available Se supplements contained one or more forms of Se, including inorganic selenite or selenate, selenomethionine, Se-methylselenocysteine or selenised yeast^(^^24^^)^. These forms differ in their impact on tissue Se concentration^(^^25^^)^. The supplementary Se intake was estimated and analysed separately for inorganic and organic supplements.

### Selenium status

A subgroup of the MoBa participants (*n* 2999) was also included in the Norwegian Environmental Biobank, a new project established by the Norwegian Institute of Public Health^(^^26^^,^^27^^)^. Inclusion criteria for the Norwegian Environmental Biobank were available whole blood, urine and plasma samples, available genetic data and available data from MoBa questionnaires 1–6 and the father questionnaire^(^^26^^,^^27^^)^. Whole blood was collected in heparin tubes in gestational weeks 17–18 and shipped by ordinary mail (unrefrigerated) in a Vacutainer for long-term freezing at a central bio-repository^(^^28^^,^^29^^)^. Storage temperature for whole blood was –20°C^(^^27^^)^. Se analyses were conducted at Lund University, Sweden, by inductive coupled plasma MS (iCAP Q; Thermo Fisher Scientific, Bremen, GmbH) equipped with a collision cell having kinetic energy discrimination and helium as collision gas. The detection limit was 3·2 µg/l, and the CV was 1·5 %. The analytical accuracy was verified using certified reference materials, Seronorm Trace elements whole blood L-1 and L-2 (SERO AS, Billingstad, Norway). The results obtained were represented as mean (standard deviation (sd)) were for L-1 (Lot 1103128, *N* 205) 56·1 (5·7) μg/l *v*. recommended 59 (35–83) μg/l and for L-2 (Lot 1103129, *N* 205) 116 (1·5) μg/l *v*. recommended 112 (66–158) μg/l^(^^27^^)^. Blood Se reflects both status and uptake while plasma/serum Se only reflects short-term status^(^^30^^)^.

Se measurements were available for 2637 of the 72 025 women included in the present study.

### Outcome variables

Gestational length in days was determined in second trimester using ultrasound in 98·2 % of the pregnancies and based on last menstrual period in the remaining pregnancies. PTD was defined as birth between week 22^+0^ and 36^+6^. Early PTD was defined as delivery between week 22^+0^ and 33^+6^, late PTD as week 34^+0^ and 36^+6^, early term as 37^+0^ and 38^+6^ and late term as deliveries after week 39^+0^. Spontaneous PTD was defined as birth after preterm labour or preterm pre-labour rupture of membranes.

### Covariates

All multivariate models with Se intake (from food or from supplements) as exposure variable were adjusted for the following pre-defined covariates: maternal age, parity, maternal smoking habits during pregnancy, alcohol consumption, maternal education, pre-pregnancy BMI, iodine intake, protein intake, fibre intake, *n*-3 intake and total energy intake. Further, models were mutually adjusted for the different Se sources (dietary intake, organic supplements, inorganic supplements). Multivariate models with Se status as exposure variable were adjusted for maternal age, parity, maternal smoking habits during pregnancy, alcohol consumption, maternal education and pre-pregnancy BMI.

Information regarding maternal age at delivery was obtained from MBRN and used as a continuous variable in the regression models. Information on parity was based on the data from both MBRN and the first MoBa questionnaire (Q1) that were distributed in pregnancy week 15–17 and divided into categories based on the number of previous pregnancies of ≥22+0 weeks of gestation: 0, 1 or >1 previous births. All other covariates were obtained from the MoBa questionnaire Q1. Maternal education was categorised as <13, 13–16, >16 years or missing. Smoking during pregnancy was categorised as non-smoker, occasional or daily smoker. Consumption of alcohol-containing beverages was self-reported in the FFQ (glasses per d, week or month) and dichotomised (yes or no) in the analyses. Pre-pregnancy BMI was based on the self-reported pre-pregnancy height and weight. BMI was grouped according to WHO classification as underweight (<18·5 kg/m^2^), normal-weight (18·5–24·9 kg/m^2^), overweight (25·0–29·9 kg/m^2^) and obese (≥30·0 kg/m^2^). Dietary fibre was included as a proxy for a healthy diet. We have previously reported that the ‘healthy/prudent dietary pattern’ correlated with fibre intake (*r* 0·57)^(^^31^^)^. The study population has been shown to be moderately iodine deficient^(^^32^^,^^33^^)^, and iodine has also been found to be associated with PTD^(^^34^^)^. As dietary iodine and Se intake correlated with *r* 0·57, and both are important for thyroid hormone production, iodine intake ranked into quintiles was included in the models. Models were also adjusted for dietary *n*-3 intake since Se and *n*-3 are both abundant in marine foods and *n*-3 intake also has been found to decrease the risk of PTD and increase pregnancy length^(^^35^^,^^36^^)^. All models were adjusted for total energy intake in kilo Joules (kJ) as well as for total protein intake since many Se sources are protein rich such as meat, poultry and fish. All variables extracted from the MoBa questionnaires were used as categorical variables in the regression models, with missing data as a category of its own.

### Statistical methods

All statistical analyses were performed using SPSS Statistics version 25.0 (International Business Machines Corp., IBM). All *P* values were two sided and values <0·05 were considered significant. Spearman’s correlation test was used to evaluate the correlation between Se intake and blood Se concentration (Table 1). Differences in Se intake and Se status according to maternal characteristics were studied with the Kruskal–Wallis test (Table 2). Multiple linear regression analysis (using the UNIANOVA command in SPSS) was used to analyse the association between Se intake or Se status and gestational length as a continuous variable (Tables 3 and 5). The association between Se intake/status and PTD was estimated as a hazard ratio (HR) with a 95 % CI using multivariable Cox regression (Tables 4 and 6). All analyses were performed with and without adjustment for the confounders listed previously. A sub-analysis was performed in order to study the association between Se intake and different categories of PTD (early preterm, late preterm and early term) (online Supplementary Table S1). In order to study the possible threshold effects between Se intake and PTD, Se intake from diet was analysed in deciles (online Supplementary Table S2). The category closest to the recommended daily intake (RDI; 60 µg/d) was used as the reference. Regarding power, in the analysis based on dietary Se intake, the group sizes are very large (3618 PTD *v*. 68 407 term deliveries) and hence even small differences can be detected. For Se status based on the subgroup with blood samples, the groups sizes are smaller (80 *v*. 2558). Here, a difference between PTD and term deliveries of 0·32 Cohen’s *d* can be detected with 80 % power at a significant level of 0·05.

**Table 1.** Correlation between selenium intake and whole-blood concentration of selenium (Numbers of subjects; medians and interquartile ranges (IQR); correlations and 95 % confidence intervals)

	Se intake (µg/d)						Correlations between intake and whole-blood concentrations					
	All subjects			Supplement users only			All subjects			Supplement users only		
	*n*	Median	IQR	*n*	Median	IQR	*n*	Rho*	95 % CI	*n*	Rho	95 % CI
Diet	72 025	53	44–62	23 409	53	44–63	2638	0·135	0·10, 0·17	914	0·115	0·05, 0·18
All supplements (organic + inorganic)	72 025	0	0–30	23 409	50	30–75	2638	0·144	0·11, 0·18	914	−0·016	−0·05, 0·05
Organic Se supplement	72 025	0	0–0	3283	30	21–43	2357	0·080	0·04, 0·12	112	0·148	−0·04, 0·32
Inorganic Se supplement	72 025	0	0–21	20 812	50	35–75	2357	0·106	0·07, 0·15	828	−0·014	−0·08, 0·05

* Spearman correlation.

**Table 2.** Maternal dietary selenium intake and maternal blood selenium concentration according to maternal characteristics† (Numbers of subjects and percentages; medians and interquartile ranges (IQR))

		Se intake from food (µg/d)					Blood Se concentration (µg/l)				
		*n*	%	Median	IQR	*P * *	*n*	%	Median	IQR	*P * *
Total		72 025	100	53	44–62		2638				
Maternal age (years)	<25	8284	12	50	41–62	<0·0001	220	8	98	86–112	<0·0001
	25–29	24 411	34	52	43–62		947	36	102	88–116	
	30–34	30 524	42	53	45–63		1180	45	102	90–118	
	>34	8806	12	54	46–64		291	11	105	91–122	
Pre-pregnancy BMI (kg/m^2^)	<18·5	2143		53	44–64	<0·0001	81	3	104	91–116	0·024
	18·5–24·9	46 163	64	53	45–63		1703	65	102	90–119	
	25–30	15 189	21	52	43–62		611	23	101	88–116	
	>30	6657	9	51	42–61		194	7	100	86–114	
	Missing	1873	3	53	44–62		49	2	101	93–113	
Parity	0	38 310	53	52	43–62	<0·0001	1515	57	104	91–119	<0·0001
	1	21 650	30	53	44–62		755	29	101	88–115	
	2	9700	13	54	45–63		317	12	100	87–115	
	3+	2306	3	54	45–64		50	2	91	81–103	
	Missing	59	0	56	45–64		1	0	112	112–112	
Preterm birth	Yes	3618	5	52	44–62	0·022	80	3	103	90–114	0·769
	No	68 407	95	53	44–62		2558	97	102	89–117	
Gestational age groups	Early preterm (GW 22–33)	952	1	51	43–61	0·026	8	0	92	81–102	0·434
	Late preterm (GW 34–36)	2666	4	53	44–62		72	3	104	93–115	
	Early term (GW 37–38)	11 660	16	53	44–63		410	16	102	89–117	
	Late term (GW 39–42)	56 747	79	53	44–62		2148	81	102	89–117	
Maternal education (years)	<13	22 424	31	51	42–62	<0·0001	677	26	100	86–114	<0·0001
	13–16	29 854	41	53	44–62		1248	47	101	88–116	
	>16	18 202	25	54	46–64		655	29	107	94–122	
	Missing	1545	2	51	42–62		58	2	98	87–115	
Smoking habits	Never	65 765	91	53	44–62	<0·0001	2462	93	102	90–118	0·001
	Occasionally	1953	3	52	43–63		63	2	99	90–113	
	Daily	3900	5	52	42–62		103	4	95	85–110	
	Missing	407	1	52	43–62		10	0	97	90–114	
Alcohol consumption	No	64 079	89	53	44–62	<0·0001	2329	88	102	89–117	0·02
	Yes	7946	11	54	46–63		309	12	104	92–120	
Iodine intake (µg/d)	<82	14 389	20	42	35–49	<0·0001	510	19	101	88–118	0·50
	82–108	14 389	20	49	42–56		554	21	103	90–118	
	108–135	14 389	20	52	45–60		523	20	102	89–117	
	135–174	14 389	20	57	50–65		554	21	103	91–117	
	>174	14 389	20	65	56–75		497	19	101	88–116	
*n*-3 intake (g/d)	<0·25	26 547	37	46	38–54	<0·0001	986	37	98	87–113	<0·0001
	0·25–0·47	24 548	34	53	46–61		977	37	105	91–118	
	>0·47	20 851	29	62	53–72		675	26	106	92–120	
Protein intake (g/d)	<76	24 008	33	42	36–48	<0·0001	868	33	102	88–118	0·79
	76–93	24 009	33	53	47–59		907	34	102	89–117	
	>93	24 008	33	65	58–74		863	32	102	90–117	
Fibre intake (g/d)	<26	24 008	33	43	37–51	<0·0001	870	33	101	88–116	0·04
	26–35	24 009	33	53	46–60		933	35	102	89–117	
	>35	24 008	33	63	55–72		835	32	104	90–118	
Energy intake (MJ/d)	<8390	24 008	33	44	37–50	<0·0001	913	35	103	90–118	0·21
	8390–10 460	24 009	33	53	46–60		893	34	102	89–117	
	>10 460	24 008	33	63	55–73		832	32	101	89–117	

GW, gestational week.

* *P* value obtained with the Mann–Whitney *U* test for two groups and the Kruskal–Wallis test for more than two groups.

† Amount of daily Se intake from food (FFQ data) and concentration of Se in blood in mid-pregnancy, according to maternal characteristics, from 72 025 participants in the Norwegian Mother and Child Cohort Study. Se intake from food was assessed with an FFQ in gestational week 22. Blood Se concentration was measured in whole blood collected in GW 17–18 in a subsample of 2638 of the 72 025 participating mothers.

**Table 3.** Association between maternal dietary selenium intake and selenium intake from supplements and gestational length* (*β*-Coefficients and 95 % confidence intervals)

	Unadjusted			Adjusted†		
	*β* ‡	95 % CI	*P*	*β* ‡	95 % CI	*P*
All subjects (*n* 72 025)						
Se intake from food§	0·07	−0·02, 0·17	0·13	0·25	0·07, 0·43	0·006
Se intake from inorganic supplements§	−0·04	−0·14, 0·05	0·35	−0·08	−0·17, 0·02	0·10
Se intake from organic supplements§	0·07	−0·03, 0·16	0·17	0·07	−0·03, 0·17	0·19
Spontaneous deliveries (*n* 57 098)						
Se intake from food§	0·13	0·03, 0·22	0·007	0·34	0·16, 0·52	1·7 × 10^−4^
Se intake from inorganic supplements§	−0·08	−0·17, 0·01	0·09	−0·09	−0·18, 0·01	0·07
Se intake from organic supplements§	0·07	−0·03, 0·16	0·17	0·07	−0·02, 0·17	0·14
Subjects with intake levels below RDI (*n* 41 581)‖						
Se intake from inorganic supplements§	−0·12	−0·24, 0·00	0·06	−0·12	−0·23, 0·02	0·05
Se intake from organic supplements§	0·08	−0·04, 0·21	0·19	0·10	−0·02, 0·21	0·10
Subjects with intake levels below 30 µg/d (*n* 1308)						
Se intake from inorganic supplements§	0·32	−0·35, 0·98	0·35	0·41	−0·25, 1·1	0·22
Se intake from organic supplements§	−0·42	−1·19, 0·35	0·28	−0·53	−1·3, 0·23	0·17

RDI, recommended daily intake.

* Multiple linear regression analysis of Se intake from food and from supplements in relation to gestational length in days for 72 025 participants in the Norwegian Mother and Child Cohort Study. Se intake from food and supplements was assessed with an FFQ in gestational week 22.

† Adjusted for maternal age, parity, smoking habits, alcohol consumption during pregnancy, maternal education, BMI, iodine intake in five categories, fibre intake, protein intake, *n*-3 intake and total energy intake. Analyses for the different Se sources are also mutually adjusted in the adjusted model.

‡ *β* per standard deviation of Se intake. Standard deviation for Se intake from food is 14·6 μg/d, from inorganic supplements 32·6 μg/d and from organic supplements 10·0 μg/d.

§ Measured in μg/d.

‖ RDI of Se for pregnant women is 60 μg/d.

**Table 4.** Association between maternal dietary selenium intake and selenium intake from supplements and risk of preterm delivery* (Hazard ratios (HR) and 95 % confidence intervals)

	Unadjusted			Adjusted†		
	HR‡	95 % CI	*P*	HR‡	95 % CI	*P*
All subjects (*n* 72 025)						
Se intake from food§	0·97	0·94, 1·00	0·06	0·92	0·87, 0·98	0·012
Se intake from inorganic supplements§	1·02	0·99, 1·05	0·21	1·01	0·98, 1·05	0·41
Se intake from organic supplements§	0·99	0·95, 1·02	0·44	0·98	0·95, 1·02	0·32
Spontaneous deliveries (*n* 57 098)‖						
Se intake from food§	0·95	0·91, 0·99	0·02	0·88	0·81, 0·96	0·003
Se intake from inorganic supplements§	1·05	1·01, 1·09	0·02	1·04	1·01, 1·03	0·08
Se intake from organic supplements§	0·99	0·94, 1·04	0·64	0·98	0·93, 1·03	0·37

* Daily intake of Se from food and from supplements and HR for preterm delivery (22^+0^–36^+6^ weeks) in all subjects and in spontaneous deliveries only analysed with Cox regression. Number of preterm deliveries: 3618 of all 72 025 subjects and 2100 of the 57 098 spontaneous deliveries. Se intake from food and supplements was assessed with an FFQ in gestational week 22.

† Adjusted for: maternal age, parity, smoking habits, alcohol consumption during pregnancy, maternal education, BMI, iodine intake in five categories, fibre intake, *n*-3 intake and total energy intake. Analyses for the different Se sources are also mutually adjusted in the adjusted model.

‡ HR per standard deviation of Se intake. Standard deviation for Se intake from food is 14·6 μg/d, from inorganic supplements 32·6 μg/d and from organic supplements 10·0 μg/d.

§ Measured in μg/d.

‖ Iatrogenic deliveries have been censored in the regression model.

**Table 5.** Association between maternal selenium blood concentration in mid-pregnancy and gestational length* (*β*-Coefficients and 95 % confidence intervals)

	Unadjusted			Adjusted†		
	*β* ‡	95 % CI	*P*	*β* ‡	95 % CI	*P*
All subjects (*n* 2638)						
Blood Se concentration§	0·12	−0·27, 0·50	0·55	−0·02	−0·41, 0·38	0·94
Spontaneous deliveries (*n* 2177)						
Blood Se concentration§	0·10	−0·28, 0·49	0·60	0·03	−0·36, 0·42	0·89

* Multiple linear regression analysis of blood Se concentrations in relation to gestational length in days for 2638 participants in the Norwegian Mother and Child Cohort Study. Blood Se concentration was measured in whole blood collected in gestational week 17−18 in a subsample of 2638 of the 72 025 participating mothers.

† Adjusted for: maternal age, parity, smoking habits, alcohol consumption during pregnancy, maternal education and BMI.

‡ *β* per standard deviation (23·4 μg/l) of blood Se concentration.

§ Measured in μg/l.

**Table 6.** Association between maternal selenium blood concentration in mid-pregnancy and risk of preterm delivery* (Hazard ratios (HR) and 95 % confidence intervals)

	Unadjusted			Adjusted†		
	HR‡	95 % CI	*P*	HR‡	95 % CI	*P*
All subjects (*n* 2638)						
Blood Se concentration§	0·91	0·72, 1·15	0·42	0·93	0·74, 1·18	0·93
Spontaneous deliveries (*n* 2177)‖						
Blood Se concentration§	0·91	0·69, 1·21	0·52	0·92	0·69, 1·22	0·56

* Maternal blood Se concentrations (μg/l) and HR for preterm delivery (22^+0^–36^+6^ weeks) in all subjects with Se blood measurements (*n* 2638) and in spontaneous deliveries only (*n* 2177) analysed with Cox regression. Number of preterm cases: 80 of all 2638 subjects and 55 of the 2177 spontaneous deliveries. Blood Se concentrations were measured in whole blood collected in gestational week 17–18.

† Adjusted for maternal age, parity, smoking habits, alcohol consumption during pregnancy, maternal education and BMI.

‡ HR per standard deviation (23·4 μg/l) of blood Se concentrations.

§ Measured in μg/l.

‖ Iatrogenic deliveries have been censored in the regression model.

### Ethical approvals

The establishment of MoBa and initial data collection was based on a license from the Norwegian Data protection agency and approval from The Regional Committee for Medical Research Ethics. The MoBa cohort is currently regulated by the Norwegian Health Registry Act. The present study was approved by The Regional Committee for Medical Research Ethics (2015/2425/Rek sør-øst A).

## Results

### Selenium intake and selenium status in the study population

**Fig. 2.**
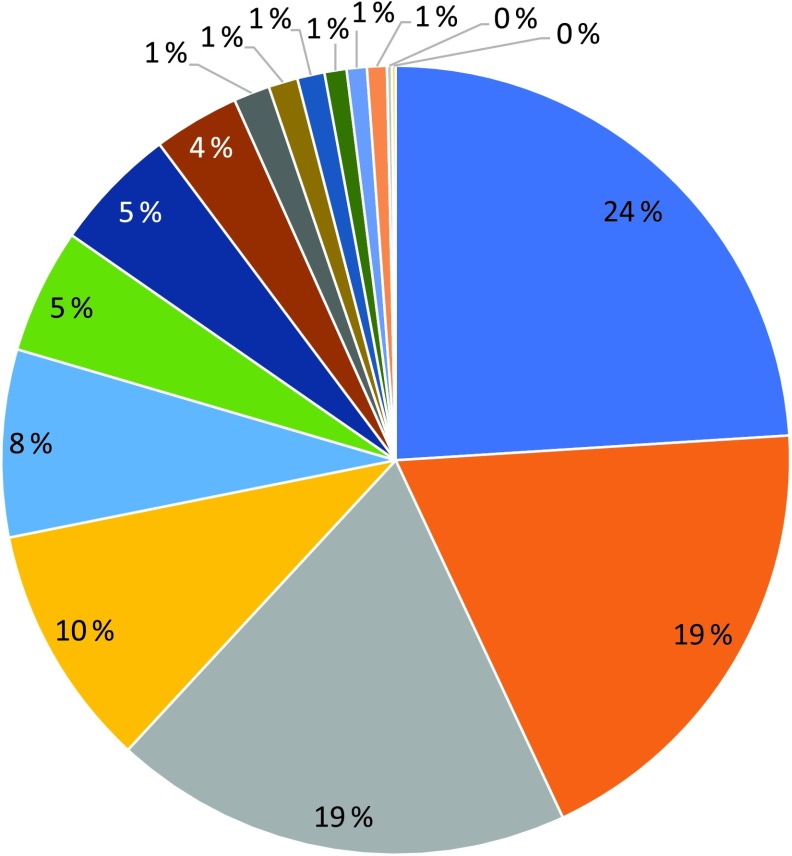
Contribution (%) to dietary selenium intake by different food sources. 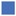, Seafood; 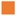, bread; 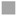, meat, all types; 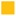, pasta; 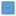, dairy products; 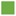, fruit and vegetables; 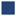, eggs; 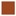, cheese; 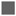, rice, millet, couscous; 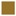, waffles, pancakes; 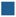, pizza, tacos; 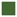, cereals; 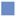, snacks and candy; 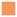, biscuits and cookies; 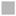, nuts; 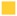, rice porridge.

The median intake of Se from the diet was 53 (interquartile range (IQR) 44–62) µg/d for all women (*n* 72 025, Table 1). Fig. 2 shows how much the different food groups contributed to dietary Se intake. Seafood, bread, meat and pasta accounted for 24, 19, 19 and 10 % of the Se in the diet, respectively. Other important sources included dairy products, fruits and vegetables, eggs and cheese (Fig. 2). In Norway, no food items are fortified with Se.

Median Se intake from supplements was 50 (IQR 30–75) µg/d for the 23 409 (33 %) Se supplement users (Table 1). Most of these women (*n* 20 812) consumed Se supplements containing only inorganic selenate or selenite, which provided a median supplemental intake of 50 (IQR 35–75) µg/d (Table 1). A smaller number (*n* 3283) took supplements containing organic selenomethionine or selenised yeast, which provided a median supplemental intake of 30 (IQR 21–43) µg/d (Table 1). The RDI of Se for pregnant women is 60 µg/d. About a third of the cohort (*n* 21 073) met the recommended dietary intake from their diet alone, while half (*n* 36 135) did so through the use of Se-containing supplements. The women with a dietary Se intake above RDI were those more likely to consume Se supplements compared with women whose Se intake is below RDI (33 *v*. 32 %, *P* = 0·005).

**Fig. 3.**
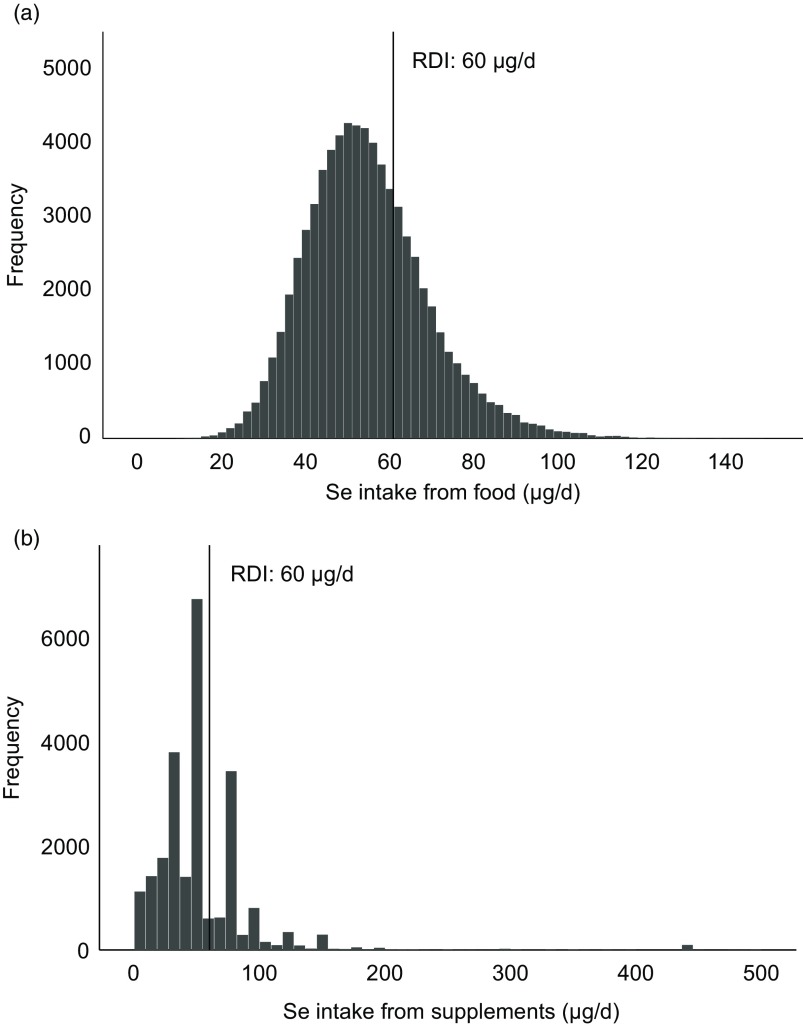
Distribution of dietary selenium intake and selenium intake from supplements (organic and inorganic forms combined). Histograms showing the intake of selenium from food (a) and from supplements (b). Only supplement users (*n* 23 409) are included in (b). RDI, recommended daily intake. For pregnant women, the RDI for selenium is 60 µg/d.

Median whole-blood Se concentration (Se status) was 102 (IQR 89–117) µg/l. This parameter correlated weakly with the estimated dietary Se intake (Spearman’s *ρ* = 0·135, *P* < 0·001); while it was not correlated with estimated Se intake from supplements (Table 1). The distribution of Se intake and whole-blood Se concentration is shown in Fig. 3.

### Maternal characteristics in relation to selenium intake and selenium status

Dietary Se intake differed by most of the maternal characteristics studied (Table 2). Dietary Se intake was positively associated with age, education, household income, parity, gestational length, alcohol consumption during pregnancy, iodine intake, protein intake, *n*-3 intake, fibre intake and total energy intake, while it was negatively associated with smoking and BMI (Table 2).

Se status was positively associated with age, education, alcohol consumption, *n*-3 intake and fibre intake and negatively associated with maternal BMI, parity and smoking (Table 2).

### Maternal selenium intake and gestational length

Dietary Se intake was positively associated with the length of gestation; *β* per sd = 0·25, adjusted model, that is, an increase in intake of Se by 1 sd, 14·6 μg/d, increased gestation by 6 h (Table 3). Regression analysis for spontaneous deliveries (*n* 57 098) only showed a positive association between maternal dietary Se intake and length of gestation (Table 3).

Among all 72 025 deliveries, 3618 (5 %) women delivered preterm, 952 (1 %) women delivered early preterm, 2666 (4 %) late preterm, 11 660 (16 %) early term and 56 747 (79 %) delivered late term. Among the 57 098 spontaneous deliveries, 2100 (4 %) were preterm, 482 (1 %) early preterm, 1618 (3 %) late preterm, 7873 (14 %) early term and 47 125 (82 %) late term.

Higher maternal dietary Se intake was associated with decreased risk of PTD in all subjects (HR per SD: 0·92, 95 % CI, 0·87, 0·98) as well as cases of spontaneous deliveries only (HR per sd: 0·88, 95 % CI, 0·81, 0·96) (Table 4).

No associations were found between intake of inorganic (selenite/selenate) or organic Se supplements (selenomethionine/selenised yeast) and gestational length or PTD (Tables 3 and 4).

Analyses of intake of dietary Se and Se-containing supplements in relation to the risk of early preterm, late preterm and early term deliveries showed one significant association, that is, Se intake from food was associated with a decreased risk of late PTD (adjusted HR per sd: 0·90, 95 % CI, 0·84, 0·97) (online Supplementary Table S1).

### Threshold analysis

Possible threshold effects of dietary Se intake were studied by entering Se categorised into deciles into the Cox regression models (online Supplementary Table S2). Women in four of the seven lowest intake categories had an increased risk of PTD compared with the reference category. No significant association was found for higher intake categories compared with the reference category (online Supplementary Table S2).

### Maternal selenium status, gestational length and preterm delivery

Maternal Se status at gestational week 17 was associated neither with gestational length nor with PTD (Tables 5 and 6). The subgroup with available whole-blood Se measurements was relatively small (*n* 2638) and included only eighty women who delivered preterm, fifty-five of these with spontaneous deliveries, limiting the statistical power.

## Discussion

Higher maternal dietary Se intake in the first half of pregnancy was associated with increased gestational length and reduced risk of PTD. The results were more pronounced when the analyses were confined to women with spontaneous deliveries only. Threshold analyses suggest that the association is driven by Se intake below the RDI and that Se intake above the RDI does not affect PTD risk. While a 6-h increase in gestational length might not seem clinically relevant, this can be expected due to the intake of a single nutrient at the population level. The corresponding findings for a decreased PTD risk underline the clinical relevance of the results. No associations were found between the intake of Se-containing supplements or Se status and gestational length or risk of PTD.

Most of the dietary Se are in proteins. The bioavailability of dietary Se depends on the digestibility of the proteins. The protein-bound forms (selenocysteine and selenomethionine) need to be converted to single amino acids and short polypeptides that can be absorbed^(^^37^^)^. Se in animal products tends to be better utilised compared with Se from plant tissues^(^^37^^,^^38^^)^ due to the better digestibility. Fish can be an exception^(^^39^^)^ if it is high in heavy metals that bind Se in poorly digestible adducts^(^^40^^)^. Dietary supplements typically contain Se in the form of Se -enriched brewers’ yeast or sodium selenite or selenate. Se in yeast needs to be digested to free Se from the yeast proteins; while the inorganic forms (sodium selenite or selenate) can be absorbed directly. Hence, bioavailability probably does not explain the different results for dietary Se being associated with gestational length, while there was no association with Se intake from supplements. Rather, the results suggest that most of the women in the cohort have dietary Se intake near or above the level associated with optimal selenoprotein expression, especially the third of women who also consumed Se-containing supplements who are more likely to have Se intake from food more than the RDI. Thus, intake of Se-containing supplements would not further improve selenoprotein expression affecting metabolic pathways related to birth outcome, for example, inflammation or redox status.

Se in whole blood circulates either bound to plasma-selenoproteins glutathione peroxidase 3 and selenprotein 1 or in the form of glutathione peroxidase 1 in the erythrocytes and lymphocytes. Also other proteins that contain Se non-specifically incorporated in lieu of methionine are found in both compartments. The relative expression level of these three selenoproteins is thought to reflect the expression of other selenoproteins in the body. The expression of selenoproteins is dependent not only on dietary Se intake but also on genetic variation in genes involved in the Se metabolism such as dimethylglycine dehydrogenase^(^^41^^)^, selenoprotein P^(^^42^^)^, the glutathione peroxidases, cytosolic glutathione peroxidase and phospholipid glutathione peroxidase^(^^43^^)^. Based on the findings from our recent genome-wide association study, where we reported genome-wide significance for one of the genes involved in the Se metabolism, the *EEFSEC* gene, we hypothesised that Se intake and/or Se status during pregnancy would be correlated with gestational length and PTD. The epidemiological findings in the present study, that dietary Se intake in the first half of pregnancy was associated with increased gestational length and reduced risk of PTD, further support this hypothesis. As a next step, we plan to perform gene–environment interaction analyses to gain further knowledge in how Se influences the length of gestation and the risk of PTD and to examine whether *EEFSEC* polymorphisms interfere with the association between dietary Se intake and whole blood Se concentrations.

Only a few other studies evaluated Se intake in relation to gestational length and/or PTD. One small nested case–control study in New Jersey, USA, including 107 women who delivered preterm and 126 control women, used a 24-h recall dietary assessment to estimate Se intake at entry to prenatal care. The results showed that women who delivered preterm had a higher Se intake in the first trimester compared with controls (115 (9) *v*. 93 (5) µg/d, *P* < 0·03)^(^^14^^)^, hence opposite results to what was found in the present study. However, the New Jersey study compared only 107 cases with 126 controls, while the present study compared 3618 cases with 68 407 controls. Further, the FFQ used in the MoBa cohort has been extensively validated^(^^18^^,^^19^^)^. A larger multicentre study in the USA, including 5738 deliveries (471 PTD cases) from the National Birth Defects Prevention Study found no association between Se intake and PTD. Se intake during the year before pregnancy was estimated retrospectively by telephone interview 6–24 months after delivery using a short FFQ of 58 food items, impairing the validity of the results^(^^13^^)^. For the MoBa cohort, dietary data were collected prospectively with an extensively validated instrument in a more than 10 times as large study population. The USA study reported a higher intake compared with that reported in Norway in the MoBa cohort^(^^13^^)^.

For 2638 of the 75 025 women included in the present study, Se status measured in gestational weeks 17–18 was available. No associations were found between Se status and either of the outcomes, gestational length and PTD. We are not aware of other studies that evaluated Se in whole blood in the first half of pregnancy in relation to gestational length and PTD. However, in accordance with the present findings, Bogden *et al*.^(^^14^^)^ observed no differences in plasma Se concentration at gestational week 15 for women who delivered preterm (*n* 107) compared with those who delivered at term (*n* 126) (1·37 (0·02) *v*. 1·34 (0·02) µmol/l). On the other hand, Rayman *et al*.^(^^15^^)^ found that women in the Netherlands who delivered preterm (*n* 60) had lower serum Se concentration at gestational week 12 than those who delivered at term (0·96 (0·14) *v*. 1·02 (0·13) μmol/l, *P* = 0·001). We have reported Se concentration in whole blood in µg while Rayman *et al*. and Bogden *et al*. reported the Se concentrations in serum in µmol. The median Se of 102 µg/l in whole blood found in our study population corresponds to 1·3 µmolar/l, which is comparable with the concentration measured in serum and plasma in the other studies.

Se from diet and supplements containing organic Se (selenomethionine or selenised yeast) was weakly correlated with Se concentration in blood, while no association was found for inorganic Se. Selenomethionine, the dominant form in foods, is incorporated specifically into selenoproteins as well as non-specifically into other proteins, for example, albumin. It is likely that women consuming less than 60 µg of the RDI had suboptimal expression of selenoenzymes, and they would be expected to show increased selenoprotein expression in response to all absorbable forms of Se.

An interesting aspect of our findings is that blood Se concentrations in Norwegians have dropped quite dramatically in the last 30 years, from being among the highest in Europe, on average approximately 150 µg/l^(^^44^^)^ whole blood (corresponding to 120 µg/l serum/plasma) to approximately 100 µg/l in the present study. This has brought Norwegians down to similar blood Se concentration as found among Swedes and Danes. This change may relate to decreasing consumption of *trans*-Atlantic flour from Se-rich areas in Canada and the USA, in favour of increased use of North-European grains, which are typically of lower Se content. The finding in the present study that supplementary Se had little influence on the blood concentration might be due to the fact that the third of women who chose to consume Se-containing supplements were those who were more likely to meet the RDI by their dietary Se intake.

### Strengths and limitations

This is by far the largest study investigating the association between Se intake and gestational length and PTD. The prospective design and the detailed information about maternal diet, demography, socioeconomic factors and pregnancy outcomes are further strengths. The study is population based, including women from all over Norway, representing women living in urban and rural areas, coastland and inland regions, different socioeconomic groups and women with diverse dietary habits. Due to the large sample size, even subgroups of PTD could be studied.

Observational studies cannot establish causality as there is always a risk of residual confounding. However, the comprehensive data set allowed us to account for a large number of possible confounders, including maternal age and education, parity, smoking and energy intake.

The dietary intake was estimated before delivery using an FFQ specifically developed and validated for use in this cohort^(^^17^^,^^19^^)^. Se intake estimated using this instrument was significantly correlated with Se intake estimated using a 4-d food diary in the validation study (*r* 0·28 for Se in food)^(^^19^^)^. However, the use of an FFQ has limitations and is more suitable for ranking participants according to high and low intake than for precise intake calculations. Since the Se content varies according to the Se concentration of the soil where crops are grown or the animals graze, some uncertainty is associated with estimating Se intake from food composition databases. When testing whether the Se content in blood varied depending on the year of inclusion into MoBa, significant differences were found, with concentrations ranging from a median of 87 to 114 µg/l between years 2003 and 2009 (results not shown). Women in the present study reported the intake of food and dietary supplements. Food habits are often stable over time, while intake of supplements fluctuate, especially during such a sensitive period as pregnancy, suggesting that the long-term intake of Se, and hence storage of Se in the body, is better captured in the questions about food than about supplement use.

Participants in MoBa were recruited between 10 and 20 years ago and dietary trends change over time. This may limit the translation of the findings. However, Se is found in many different food items and most of them are staple foods such as wheat, meat and fish. We have little reason to believe that major changes were observed in the intake pattern of Se-rich food items during the past decade, though the dishes they are part of may have changed.

The generalisability of the results from the Se measurements in whole blood may be limited due to selection bias. Since one inclusion criterion for participation in the Norwegian Environmental Biobank Study was to have answered all of the first six MoBa questionnaires, the subgroup of women who have Se measurements do not represent the whole MoBa population^(^^27^^)^. Women in the subsample included a lower proportion with missing information on baseline characteristics and a higher proportion of non-smokers and highly educated compared with those in the whole MoBa^(^^27^^)^.

### Conclusions

Higher Se intake from food but not from supplements was associated with a small increase in gestational length and decreased risk of PTD in 72 075 women participating in MoBa, strengthening the results from the first genome-wide association study on gestational length and PTD^(^^4^^)^. The reason for the different findings for Se in different sources, that is, from supplements and from food, may be due to the fact that about half of the women had a dietary Se intake more than the RDI, which is thought to guarantee an optimal selenoenzyme expression. Further studies, preferably in the form of randomised controlled trials and gene–environment interaction analyses, are needed before considering to change the present dietary guidelines regarding Se intake by pregnant women.

## Supplementary material

For supplementary material accompanying this paper visit https://doi.org/10.1017/S0007114519002113. click here to view supplementary material

## References

[1] Morken NH , Vogel I , Kallen K , et al (2008) Reference population for international comparisons and time trend surveillance of preterm delivery proportions in three countries. BMC Womens Health 8, 16. 18817549 10.1186/1472-6874-8-16 PMC2566973

[2] Ferrero DM , Larson J , Jacobsson B , et al (2016) Cross-country individual participant analysis of 4.1 million singleton births in 5 countries with very high human development index confirms known associations but provides no biologic explanation for 2/3 of all preterm births. *PLOS ONE* 11, e0162506. 10.1371/journal.pone.0162506 PMC5021369 27622562

[3] Chang HH , Larson J , Blencowe H , et al (2013) Preventing preterm births: analysis of trends and potential reductions with interventions in 39 countries with very high human development index. Lancet 381, 223–234. 23158883 10.1016/S0140-6736(12)61856-X PMC3572865

[4] Zhang G , Feenstra B , Bacelis J , et al (2017) Genetic associations with gestational duration and spontaneous preterm birth. N Engl J Med 377, 1156–1167. 28877031 10.1056/NEJMoa1612665 PMC5561422

[5] Labunskyy VM , Hatfield DL & Gladyshev VN (2014) Selenoproteins: molecular pathways and physiological roles. Physiol Rev 94, 739–777. 24987004 10.1152/physrev.00039.2013 PMC4101630

[6] Mullur R , Liu Y-Y & Brent GA (2014) Thyroid hormone regulation of metabolism. Physiol Rev 94, 355–382. 24692351 10.1152/physrev.00030.2013 PMC4044302

[7] Burnum KE , Hirota Y , Baker ES , et al (2012) Uterine deletion of Trp53 compromises antioxidant responses in the mouse decidua. Endocrinology 153, 4568–4579. 22759378 10.1210/en.2012-1335 PMC3423619

[8] Goldenberg RL , Culhane JF , Iams JD , et al (2008) Epidemiology and causes of preterm birth. Lancet 371, 75–84. 18177778 10.1016/S0140-6736(08)60074-4 PMC7134569

[9] Muglia LJ & Katz M (2010) The enigma of spontaneous preterm birth. N Engl J Med 362, 529–535. 20147718 10.1056/NEJMra0904308

[10] Chen C-Y , Chen C-P & Lin K-H (2015) Biological functions of thyroid hormone in placenta. Int J Mol Sci 16, 4161–4179. 25690032 10.3390/ijms16024161 PMC4346950

[11] World Health Organization (2012) Born Too Soon: The Global Action Report on Preterm Birth. Geneva: WHO.

[12] Hurst R , Siyame EW , Young SD , et al (2013) Soil-type influences human selenium status and underlies widespread selenium deficiency risks in Malawi. Sci Rep 3, 1425. 23478344 10.1038/srep01425 PMC3594796

[13] Carmichael SL , Yang W , Shaw GM , et al (2013) Maternal dietary nutrient intake and risk of preterm delivery. Am J Perinat 30, 579–588. 10.1055/s-0032-1329686 PMC4041277 23208764

[14] Bogden JD , Kemp FW , Chen X , et al (2006) Low-normal serum selenium early in human pregnancy predicts lower birth weight. Nutr Res 26, 497–502.

[15] Rayman MP , Wijnen H , Vader H , et al (2011) Maternal selenium status during early gestation and risk for preterm birth. CMAJ 183, 549–555. 21324870 10.1503/cmaj.101095 PMC3060183

[16] Magnus P , Birke C , Vejrup K , et al (2016) Cohort profile update: the Norwegian mother and child cohort study (MoBa). Int J Epidemiol 45, 382–388. 27063603 10.1093/ije/dyw029

[17] Meltzer HM , Brantsaeter AL , Ydersbond TA , et al (2008) Methodological challenges when monitoring the diet of pregnant women in a large study: experiences from the Norwegian Mother and Child Cohort Study (MoBa). Matern Child Nutr 4, 14–27. 18171404 10.1111/j.1740-8709.2007.00104.x PMC6860710

[18] Brantsaeter AL , Haugen M , Thomassen Y , et al (2010) Exploration of biomarkers for total fish intake in pregnant Norwegian women. Public Health Nutr 13, 54–62. 19490733 10.1017/S1368980009005904

[19] Brantsaeter AL , Haugen M , Alexander J , et al (2008) Validity of a new food frequency questionnaire for pregnant women in the Norwegian Mother and Child Cohort Study (MoBa). Matern Child Nutr 4, 28–43. 18171405 10.1111/j.1740-8709.2007.00103.x PMC6860878

[20] Lauritsen J (1998) *FoodCalc v. 1.3*: Diet, Cancer and Health Project. Copenhagen: Danish Cancer Society.

[21] Rimestad A , Borgejordet Å , Vesterhus K , et al (2005) Den store matvaretabellen (The Norwegian Food Table). Oslo: Statens råd for ernæring og fysisk aktivitet, Statens næringsmiddeltilsyn, Universitetet i Oslo ‐ Institutt for ernæringsforskning.

[22] Haugen M , Brantsaeter AL , Alexander J , et al (2008) Dietary supplements contribute substantially to the total nutrient intake in pregnant Norwegian women. Ann Nutr Metab 52, 272–280. 18645244 10.1159/000146274 PMC2813797

[23] Brantsaeter AL , Haugen M , Hagve TA , et al (2007) Self-reported dietary supplement use is confirmed by biological markers in the Norwegian Mother and Child Cohort Study (MoBa). Ann Nutr Metab 51, 146–154. 17536192 10.1159/000103275

[24] Niedzielski P , Rudnicka M , Wachelka M , et al (2016) Selenium species in selenium fortified dietary supplements. Food Chem 190, 454–459. 26212996 10.1016/j.foodchem.2015.05.125

[25] Sigrist M , Brusa L , Campagnoli D , et al (2012) Determination of selenium in selected food samples from Argentina and estimation of their contribution to the Se dietary intake. Food Chem 134, 1932–1937. 23442641 10.1016/j.foodchem.2012.03.116

[26] Paltiel L , Haugan A , Skjerden T , et al (2014) The biobank of the Norwegian mother and child cohort study – Present status. Norsk Epidemiol 24, 29–35.

[27] Caspersen IH , Thomsen C , Haug LS , et al (2019) Patterns and dietary determinants of essential and toxic elements in blood measured in mid-pregnancy: the Norwegian Environmental Biobank. Sci Total Environ 671, 299–308. 30928759 10.1016/j.scitotenv.2019.03.291

[28] Ronningen KS , Paltiel L , Meltzer HM , et al (2006) The biobank of the Norwegian Mother and Child Cohort Study: a resource for the next 100 years. Eur J Epidemiol 21, 619–625. 17031521 10.1007/s10654-006-9041-x PMC1820840

[29] Paltiel L , Anita H , Skjerden T , et al (2014) The biobank of the Norwegian Mother and Child Cohort Study – present status. Norsk Epidemiol 24, 29–35.

[30] Thomson CD (2004) Assessment of requirements for selenium and adequacy of selenium status: a review. Eur J Clin Nutr 58, 391–402. 14985676 10.1038/sj.ejcn.1601800

[31] Englund-Ögge L , Brantsæter AL , Sengpiel V , et al (2014) Maternal dietary patterns and preterm delivery: results from large prospective cohort study. BMJ 348, g1446. 24609054 10.1136/bmj.g1446 PMC3942565

[32] Abel MH , Korevaar T , Erlund I , et al (2018) Iodine intake is associated with thyroid function in mild- to moderately iodine deficient pregnant women. Thyroid 28, 1359–1371. 30132420 10.1089/thy.2018.0305 PMC6157349

[33] Abel MH , Ystrom EA-O , Caspersen IA-O , et al (2017) Maternal iodine intake and offspring attention-deficit/hyperactivity disorder: results from a large prospective cohort study. Nutrients 9, 1239. 10.3390/nu9111239 PMC5707711 29137191

[34] Charoenratana C , Leelapat P , Traisrisilp K , et al (2016) Maternal iodine insufficiency and adverse pregnancy outcomes. Matern Child Nutr 12, 680–687. 26332721 10.1111/mcn.12211 PMC6860088

[35] Middleton P , Gomersall JC , Gould JF , et al (2018) Omega-3 fatty acid addition during pregnancy. *Cochrane Database Syst Rev*, issue 11, CD003402. 10.1002/14651858.CD003402.pub3 PMC6516961 30480773

[36] Vinding RK , Stokholm J , Sevelsted A , et al (2019) Fish oil supplementation in pregnancy increases gestational age, size for gestational age, and birth weight in infants: a randomized controlled trial. J Nutr 149, 628–634. 30418579 10.1093/jn/nxy204

[37] Combs GF & Combs SB (1986) The biological availability of selenium in foods and feeds In The Role of Selenium in Nutrition, chapter 4, pp. 127–177 [ GF Combs and SB Combs , editors]. Cambridge, MA: Academic Press.

[38] Cantor AH , Moorhead PD & Musser MA (1981) Biological availability of selenium in selenium compounds and feed ingredients. In Selenium in Biology and Medicine, chapter 17, pp. 192–208 [JE Spallholz, JL Martin and HE Ganther, editors]. Westport, CT: AVI Publishing Co.

[39] Cantor AH , Scott ML & Noguchi T (1975) Biological availability of selenium in feedstuffs and selenium compounds for prevention of exudative diathesis in chicks. J Nutr 105, 96–105.

[40] Shamberger R (2012) Biological interactions of selenium and other substances In Biochemistry of Selenium, pp. 125–166. New York: Springer US, Plenum Press.

[41] Evans DM , Zhu G , Dy V , et al (2013) Genome-wide association study identifies loci affecting blood copper, selenium and zinc. Hum Mol Genet 22, 3998–4006. 23720494 10.1093/hmg/ddt239 PMC3766178

[42] Meplan C , Crosley LK , Nicol F , et al (2007) Genetic polymorphisms in the human selenoprotein P gene determine the response of selenoprotein markers to selenium supplementation in a gender-specific manner (the SELGEN study). FASEB J 21, 3063–3074. 17536041 10.1096/fj.07-8166com

[43] Meplan C , Crosley LK , Nicol F , et al (2008) Functional effects of a common single-nucleotide polymorphism (GPX4c718t) in the glutathione peroxidase 4 gene: interaction with sex. Am J Clin Nutr 87, 1019–1027. 18400727 10.1093/ajcn/87.4.1019

[44] Meltzer HM , Norheim G , Bibow K , et al (1990) The form of selenium determines the response to supplementation in a selenium replete population. Eur J Clin Nutr 44, 435–446. 2387279

