# Supplementary material for: Maternal dietary selenium intake is associated with increased gestational length and decreased risk of preterm delivery
Source: Br J Nutr. 2019 Dec 23;123(2):209–19. doi: 10.1017/S0007114519002113 (PMC7015879; doi:10.1017/S0007114519002113)
Supplement: Supplementary file 1 [file S0007114519002113sup.zip › S0007114519002113sup001.docx]

**Supplementary table 1: Association between maternal selenium intake from food and from supplements in mid pregnancy and risk for early preterm, late preterm, and early term delivery,  n=72,025 women in the Norwegian Mother, Father and Child Cohort Study (MoBa).**

|  | Unadjusted | | |  | Adjusted^1^ | | |
| --- | --- | --- | --- | --- | --- | --- | --- |
|  | HR^2^ | 95% CI of HR^2^ | P |  | HR^2^ | 95% CI of HR^2^ | P |
| **Early preterm delivery (cases=952)** |  |  |  |  |  |  |  |
| Selenium intake from food^3^ | 0.93 | 0.87 – 0.99 | 0.03 |  | 0.98 | 0.87 – 1.11 | 0.76 |
| Selenium intake from inorganic supplements^3^ | 1.02 | 0.96 – 1.08 | 0.60 |  | 1.01 | 0.95 – 1.08 | 0.80 |
| Selenium intake from organic supplements^3^ | 0.97 | 0.90 – 1.05 | 0.43 |  | 0.97 | 0.90 – 1.04 | 0.39 |
| **Late preterm delivery (cases=2,666)** |  |  |  |  |  |  |  |
| Selenium intake from food^3^ | 0.98 | 0.95 – 1.02 | 0.37 |  | 0.90 | 0.84 – 0.97 | 0.007 |
| Selenium intake from inorganic supplements^3^ | 1.02 | 0.99 – 1.06 | 0.25 |  | 1.02 | 0.98 – 1.06 | 0.42 |
| Selenium intake from organic supplements^3^ | 0.99 | 0.95 – 1.03 | 0.67 |  | 0.99 | 0.95 – 1.03 | 0.51 |
| **Early term delivery (cases=11,666)** |  |  |  |  |  |  |  |
| Selenium intake from food^3^ | 1.00 | 0.98 – 1.01 | 0.63 |  | 0.98 | 0.94 – 1.01 | 0.19 |
| Selenium intake from inorganic supplements^3^ | 1.04 | 0.99 – 1.02 | 0.69 |  | 1.01 | 0.99 – 1.03 | 0.22 |
| Selenium intake from organic supplements^3^ | 0.99 | 0.97 – 1.01 | 0.39 |  | 0.99 | 0.97 – 1.01 | 0.37 |

Daily maternal dietary intake of selenium from food and from supplements and hazard ratios (HR) for early preterm delivery (22^+0^-33^+6^ weeks, n=952), late preterm delivery (34^+0^-36^+6^ weeks, n=2,666), early term (37^+0^-38^+6^ weeks, n=11,660).

Selenium intake from food and from supplements were assessed with a food frequency questionnaire in gestational week 22.

^1^Adjusted for: maternal age, parity, smoking habits, alcohol consumption during pregnancy, maternal education, BMI, iodine intake in five categories, protein intake, fiber intake, omega-3 intake and total energy intake. Analyses for the different selenium sources are also mutually adjusted in the adjusted model.

^2^HR per standard deviation of selenium intake. Standard deviations for selenium intake from food is 14.6 µg/day, from inorganic supplements is 32.6 µg/day and from organic supplements 10.0 µg/day

^3^Measured in µg/day
