# Supplementary material for: Maternal dietary selenium intake is associated with increased gestational length and decreased risk of preterm delivery
Source: Br J Nutr. 2019 Dec 23;123(2):209–19. doi: 10.1017/S0007114519002113 (PMC7015879; doi:10.1017/S0007114519002113)
Supplement: Supplementary file 1 [file S0007114519002113sup.zip › S0007114519002113sup002.docx]

**Supplementary Table 2: Threshold analyses of maternal selenium intake from food and risk for preterm delivery, n=72,025 women in the Norwegian Mother, Father and Child Cohort Study (MoBa).**

|  |  |  | Unadjusted | | | Adjusted^1^ | | |
| --- | --- | --- | --- | --- | --- | --- | --- | --- |
| Category | Mean selenium intake (µg/day) | Number of preterm cases/total subjects | HR^2^ | 95% CI of HR^2^ | P | HR^2^ | 95% CI of HR ^2^ | P |
| 1 | 31 | 373/6677 | 1.23 | 1.10 – 1.50 | 0.001 | 1.32 | 1.10 – 1.59 | 0.004 |
| 2 | 40 | 448/8516 | 1.21 | 1.04 – 1.40 | 0.01 | 1.25 | 1.06 – 1.48 | 0.008 |
| 3 | 44 | 280/5617 | 1.14 | 0.97 – 1.34 | 0.12 | 1.18 | 1.00 – 1.41 | 0.06 |
| 4 | 48 | 424/8266 | 1.17 | 1.01 – 1.36 | 0.04 | 1.22 | 1.05 – 1.42 | 0.01 |
| 5 | 51 | 290/6450 | 1.02 | 0.90 – 1.20 | 0.79 | 1.06 | 0.90 – 1.25 | 0.48 |
| 6 | 54 | 444/8278 | 1.23 | 1.06 – 1.43 | 0.006 | 1.26 | 1.09 – 1.46 | 0.002 |
| 7 | 58 | 350/7148 | 1.12 | 0.96 – 1.31 | 0.16 | 1.14 | 0.98 – 1.32 | 0.10 |
| 8 (ref^3^) | 63 | 319/7245 |  |  |  |  |  |  |
| 9 | 69 | 329/6462 | 1.17 | 1.00 – 1.36 | 0.06 | 1.13 | 0.96 – 1.31 | 0.14 |
| 10 | 83 | 361/7366 | 1.12 | 0.96 – 1.30 | 0.15 | 1.00 | 0.84 – 1.18 | 0.96 |

Daily dietary intake of selenium from food divided into tentiles and hazard ratios for preterm delivery (22^+0^-36^+6^ weeks). Number of preterm cases: 3618 of all 72,025 subjects. Selenium intake were assessed with a food frequency questionnaire in gestational week 22.

^1^Adjusted for: maternal age, parity, smoking habits, alcohol consumption during pregnancy, maternal education, BMI, iodine intake in four categories, fiber intake, protein intake, omega-3 intake, total energy intake and selenium intake from supplements.

^2^HR per standard deviation of selenium intake, i.e. 14.6 µg/day,

^3^The category with mean intake closest to the recommended daily intake (RDI) of selenium for pregnant women (60 µg/day) is used as the reference category.
